# Mapping QTLs for 1000-grain weight and genes controlling hull type using SNP marker in Tartary buckwheat (*Fagopyrum tataricum*)

**DOI:** 10.1186/s12864-021-07449-w

**Published:** 2021-02-27

**Authors:** Tao-Xiong Shi, Rui-Yuan Li, Ran Zheng, Qing-Fu Chen, Hong-You Li, Juan Huang, Li-Wei Zhu, Cheng-Gang Liang

**Affiliations:** 1 Research Center of Buckwheat Industry Technology, Guizhou Normal University, Guiyang, 550001 Guizhou China; 2 Key Laboratory of Information and Computing Science of Guizhou Province, Guizhou Normal University, Guiyang, 550001 Guizhou China

**Keywords:** Tartary buckwheat, RAD sequencing, Genetic map, Hull type, 1000-grain wight, QTLs mapping

## Abstract

**Background:**

Tartary buckwheat (*Fagopyrum tataricum*), an important pseudocereal crop, has high economic value due to its nutritional and medicinal properties. However, dehulling of Tartary buckwheat is difficult owing to its thick and tough hull, which has greatly limited the development of the Tartary buckwheat processing industry. The construction of high-resolution genetic maps serves as a basis for identifying quantitative trait loci (QTLs) and qualitative trait genes for agronomic traits. In this study, a recombinant inbred lines (XJ-RILs) population derived from a cross between the easily dehulled Rice-Tartary type and Tartary buckwheat type was genotyped using restriction site-associated DNA (RAD) sequencing to construct a high-density SNP genetic map. Furthermore, QTLs for 1000-grain weight (TGW) and genes controlling hull type were mapped in multiple environments.

**Results:**

In total, 4151 bin markers comprising 122,185 SNPs were used to construct the genetic linkage map. The map consisted of 8 linkage groups and covered 1444.15 cM, with an average distance of 0.35 cM between adjacent bin markers. Nine QTLs for TGW were detected and distributed on four loci on chromosome 1 and 4. A major locus detected in all three trials was mapped in 38.2–39.8 cM region on chromosome 1, with an LOD score of 18.1–37.0, and explained for 23.6–47.5% of the phenotypic variation. The genes controlling hull type were mapped to chromosome 1 between marker Block330 and Block331, which was closely followed by the major locus for TGW. The expression levels of the seven candidate genes controlling hull type present in the region between Block330 and Block336 was low during grain development, and no significant difference was observed between the parental lines. Six non-synonymous coding SNPs were found between the two parents in the region.

**Conclusions:**

We constructed a high-density SNP genetic map for the first time in Tartary buckwheat. The mapped major loci controlling TGW and hull type will be valuable for gene cloning and revealing the mechanism underlying grain development and easy dehulling, and marker-assisted selection in Tartary buckwheat.

**Supplementary Information:**

The online version contains supplementary material available at 10.1186/s12864-021-07449-w.

## Background

Tartary buckwheat (*Fagopyrum tataricum*) is a major cultivated species of buckwheat that is widely cultivated in the mountainous regions of Southwest Asia [1]. Tartary buckwheat strikingly differs in yield characteristics and quality parameters from common buckwheat (*Fagopyrum esculentum*), another major cultivated species of buckwheat. Tartary buckwheat has a higher grain yield due to its self-compatibility and high grain setting rate [2]. Despite its lower 1000-grain weight (TGW), Tartary buckwheat grains have higher levels of total flavonoids [3–5], crude fibre, minerals (K, Mg, Zn, Cu and Mn) [6, 7], vitamins (B1, B2, and B6) [8, 9], high-quality protein [8, 10] and antioxidant capacity [4, 11] in comparison with common buckwheat. In recent years, Tartary buckwheat has increasingly received attention due to its nutritional, economic, and pharmaceutical value. However, almost all Tartary buckwheat grains are extremely difficult to dehull owing to their thick and adherent hull with three grooves, which greatly limits the development of the Tartary buckwheat processing industry. Developing Tartary buckwheat varieties with easy dehulling is regarded as the key to solving this problem.

Rice-Tartary is a particular Tartary buckwheat type derived from a cross between wild *F. esculentum* and cultivated *F. tataricum* [12], also called Miqiao in southwest regions of China. Unlike Tartary buckwheat, Rice-Tartary grains have thin and loose hull but lack grooves and can be readily dehulled [13]. Despite the ease of dehulling, Rice-Tartary has not been widely cultivated in recent years because the plants have a long vegetative period and lower yields than those of Tartary buckwheat when grown in low-altitude areas and under long-day conditions [14]. To develop cultivars with easy dehulling and high yields, hybridization between Tartary buckwheat and Rice-Tartary was conducted [13, 15–19]. The study of the inheritance of hull types based on progeny analysis showed that the Rice-Tartary type is recessive to the Tartary buckwheat type and is controlled by a single gene [13, 16, 17]. The characteristics of dehulling have indicated to be related to grain shell thickness. The varieties with grain shell thickness > 0.20 mm and grain shell rate > 20% are thick shelled and difficult to dehull, while those with seed shell thickness < 0.15 mm and grain shell rate < 20% are thin shelled and easily dehulled [18]. Song et al. [20] investigated the relation between dehulling efficiency and content of lignin and cellulose in the mature grain hull and found that Rice-Tartary variety showed the highest content in lignin (35%) and the lowest content in cellulose compared with Tartary buckwheat varieties. According to the analysis of mechanical parameters of cytoskeleton and cell wall content, Liu et al. [21] speculated that high brittleness and high pectin content may cause the Rice-Tartary fruit to be easily cracked and dehulled and found that *FtpinG0009028000.01* gene has the potential effect on the cracking of Tartary buckwheat fruit. Fukuie et al. [22] found that the thin hull of Rice-Tartary plants is due to the lack of periclinal cell divisions underneath the epidermis in the proximity of the ovary midribs, while such periclinal cell divisions are initiated at an early stage of ovary development in Tartary buckwheat cultivars, which promotes thickening of the secondary cell wall and cell adhesion. This lack of periclinal cell division in Rice-Tartary plants is associated with a G → A substitution in *FtAG*, suggesting that *FtAG* is a candidate gene for associated with the ease of dehulling in Tartary buckwheat [22]. By combining bulked segregant analysis (BSA) and high-throughput sequencing, Zhang et al. [23] identified a candidate genetic region associated with the non-adherent hull of Rice-Tartary, containing 45 high-impact single-nucleotide polymorphisms (SNPs)/indels and 36 genes.

The gene underlying easy dehulling has not been identified until now. A genetic linkage map is an important basis for mapping qualitative trait loci and quantitative trait loci (QTLs) and identifying candidate genes for target traits. In the present study, we used a recombinant inbred lines (XJ-RILs) population derived from a cross between an easily dehulled Rice-Tartary variety and a Tartary buckwheat variety to construct a high-density linkage map using SNP markers generated by restriction site-associated DNA (RAD) sequencing. Using the high-density SNP linkage map, we mapped QTLs for TGW and genes controlling hull type in multiple environments. The identified major and reliable loci controlling TGW and hull type will be valuable for marker-assisted selection breeding, cloning the gene and studying the mechanism underlying grains development and easy dehulling of Tartary buckwheat.

## Results

### SNP genotyping based on RAD sequencing

To construct the high-density linkage map, the XJ-RILs population derived from a cross between Rice-Tartary variety “Xiaomiqiao” and Tartary buckwheat variety “Jinqiaomai 2” along with the parents was re-sequenced by an Illumina HiSeq2500 platform. Whole-genome re-sequencing produced a total of 10.05 G clean bases with 18.0-fold depth for Xiaomiqiao and 10.21 G clean bases with 21.0-fold depth for Jinqiaomai 2. In total, 300.25 G clean reads were generated for the 221 RILs by RAD sequencing, with an approximately 2.76-fold depth for each line (Additional file 1: Table S1). In total, 405,646 SNPs were identified by analysing the parental lines (Additional file 2: Table S2). All of the SNPs in the RILs were clustered in recombination bins (Additional file 3: Fig.S1). After filtration of bins with length < 15 kb and bins with an extreme segregation distortion (*P* < 0.01) by the χ^2^ test, 4151 recombination bin markers were retained to construct the genetic linkage map (Table 1, Additional file 4: Table S3).

**Table 1:** Distribution of genetic markers on the high-density genetic map

Linkage group	Number of SNP markers	Total Bin Marker	Total Distance (cM)	Average Distance (cM)	Max Gap (cM)	Gaps< 5 cM (%)
Chr.1	22,236	727	209.59	0.29	6.71	99.7%
Chr.2	20,629	511	99.03	0.19	3.62	100%
Chr.3	13,986	506	191.54	0.38	3.46	95.6%
Chr.4	18,092	515	195.24	0.38	3.67	100%
Chr. 5	20,141	561	177.53	0.32	3.39	100%
Chr.6	9873	469	178.35	0.38	2.78	100%
Chr.7	9434	451	193.01	0.43	3.65	100%
Chr.8	7794	411	199.87	0.49	11.97	93.9%
Total	122,185	4151	1444.15	0.35	–	98.7%

### Construction of the SNP genetic linkage map

**Fig. 1:**
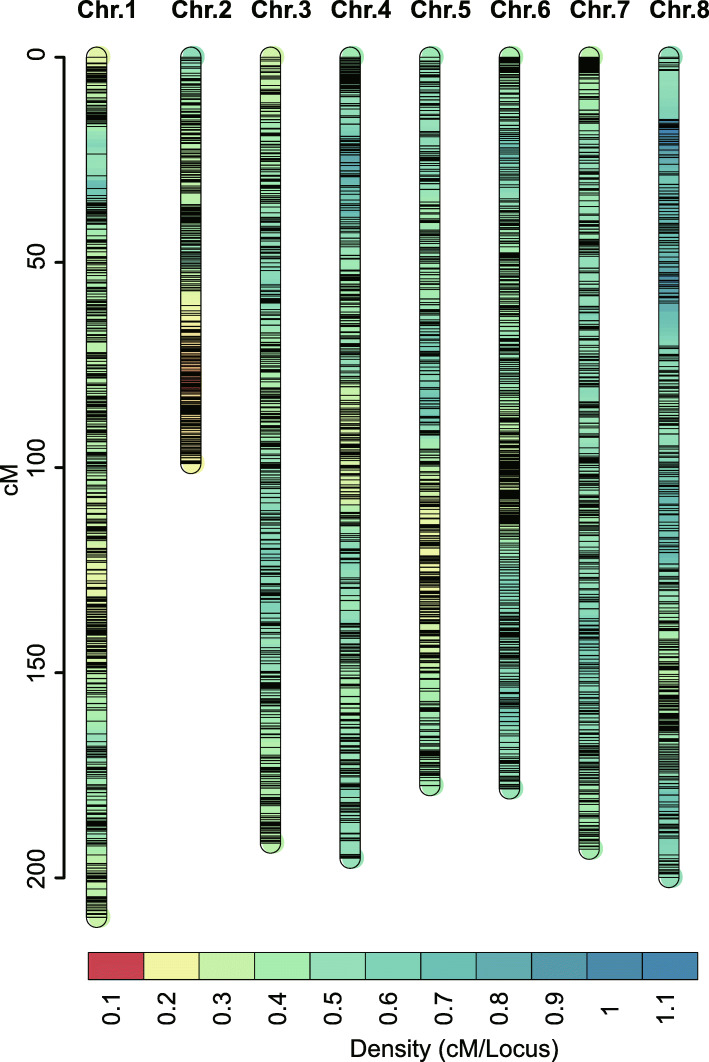
High-density genetic map of the XJ-RILs population derived from the cross of ‘Xiaomiqiao × Jinqiaomai 2’ constructed by bin markers

The constructed linkage map of Tartary buckwheat consisted of 8 linkage groups and covered 4151 bin markers comprising 122,185 SNPs, which spanned 1444.15 cM, with an average distance of 0.35 cM between adjacent markers (Table 1, Fig. 1 and Additional file 5: Table S4). Chr.1 was the longest and largest linkage group, with a genetic distance of 209.59 cM and 727 bin markers, whereas Chr.2 was the shortest linkage group, spanning 99.03 cM and containing 511 bin markers. In general, the bin markers were well distributed on the 8 linkage groups, and approximately 98.7% of the intervals between adjacent markers were less than 5 cM (Table 1).

### Collinearity of the genetic and physical maps

**Fig. 2:**
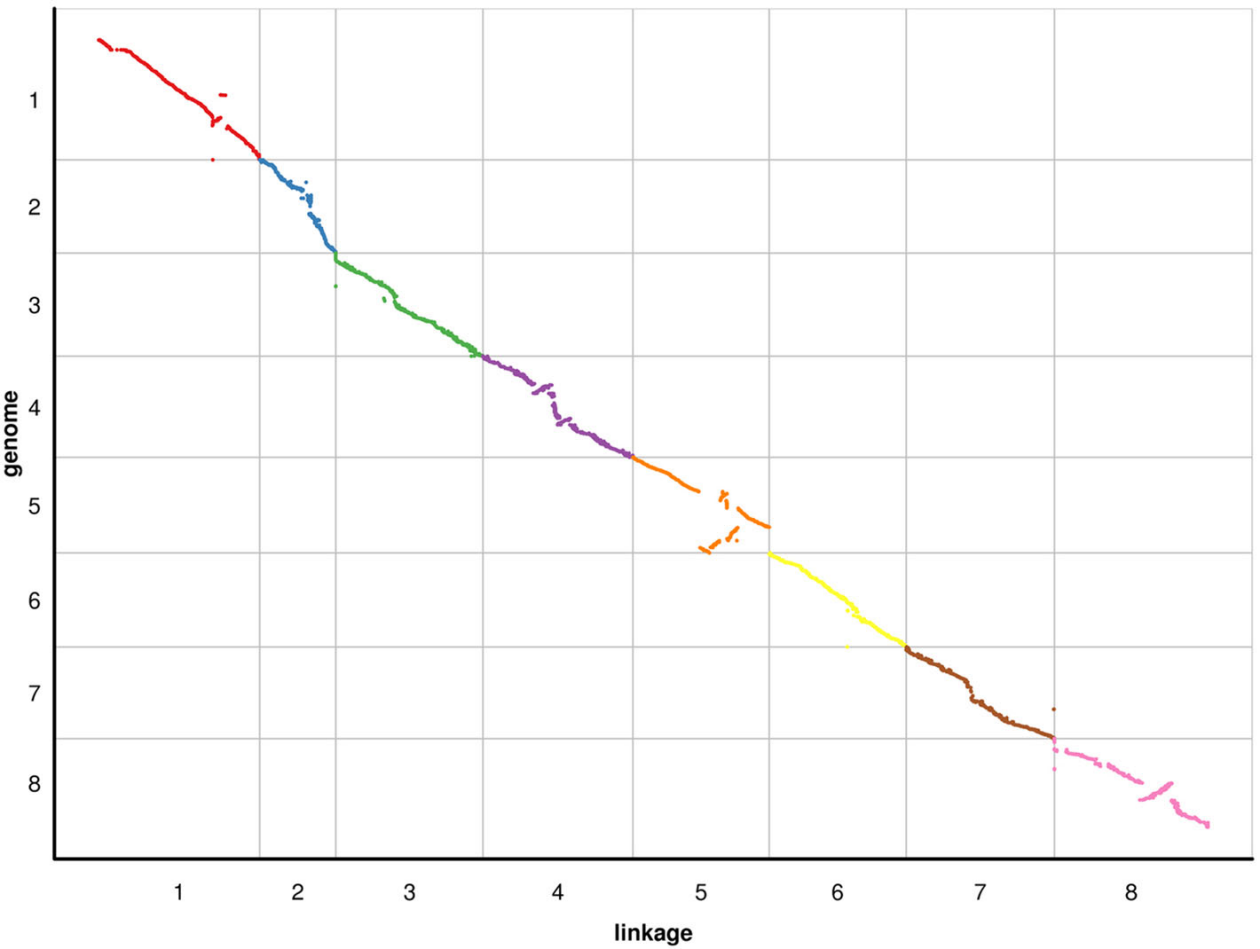
Collinearity between the genetic map derived from the XJ-RILs population derived from the cross of ‘Xiaomiqiao × Jinqiaomai 2’ and the reference genome (Pinku1). In each plot, the genetic position of the 8 linkage groups from the XJ-RILs population map is on the x-axis, and the physical positions of the 8 Tartary buckwheat chromosomes is on the y-axis

The collinearity between the genetic map and the Pinku1 Tartary buckwheat reference genome [24] was evaluated. As shown in Fig. 2, the relationships between the genetic and physical maps were generally linear for the 8 chromosomes, except for linkage group Chr.5. The Spearman correlation coefficient between the genetic and physical positions of each linkage group ranged from 0.605 to 0.997 with average of 0.94 (Table 2). These results indicated that the genetic maps have high levels of collinearity with the physical map and sufficiently cover the Tartary buckwheat genome.

**Table 2:** Spearman correlation coefficients between the genetic and physical positions of each linkage group

Linkage group	Spearman
Chr.1	0.994
Chr.2	0.997
Chr.3	0.994
Chr.4	0.997
Chr. 5	0.605
Chr.6	0.997
Chr.7	0.993
Chr.8	0.971

### Phenotype test of hull type and variation analysis of TGW in XJ-RILs population

**Fig. 3:**
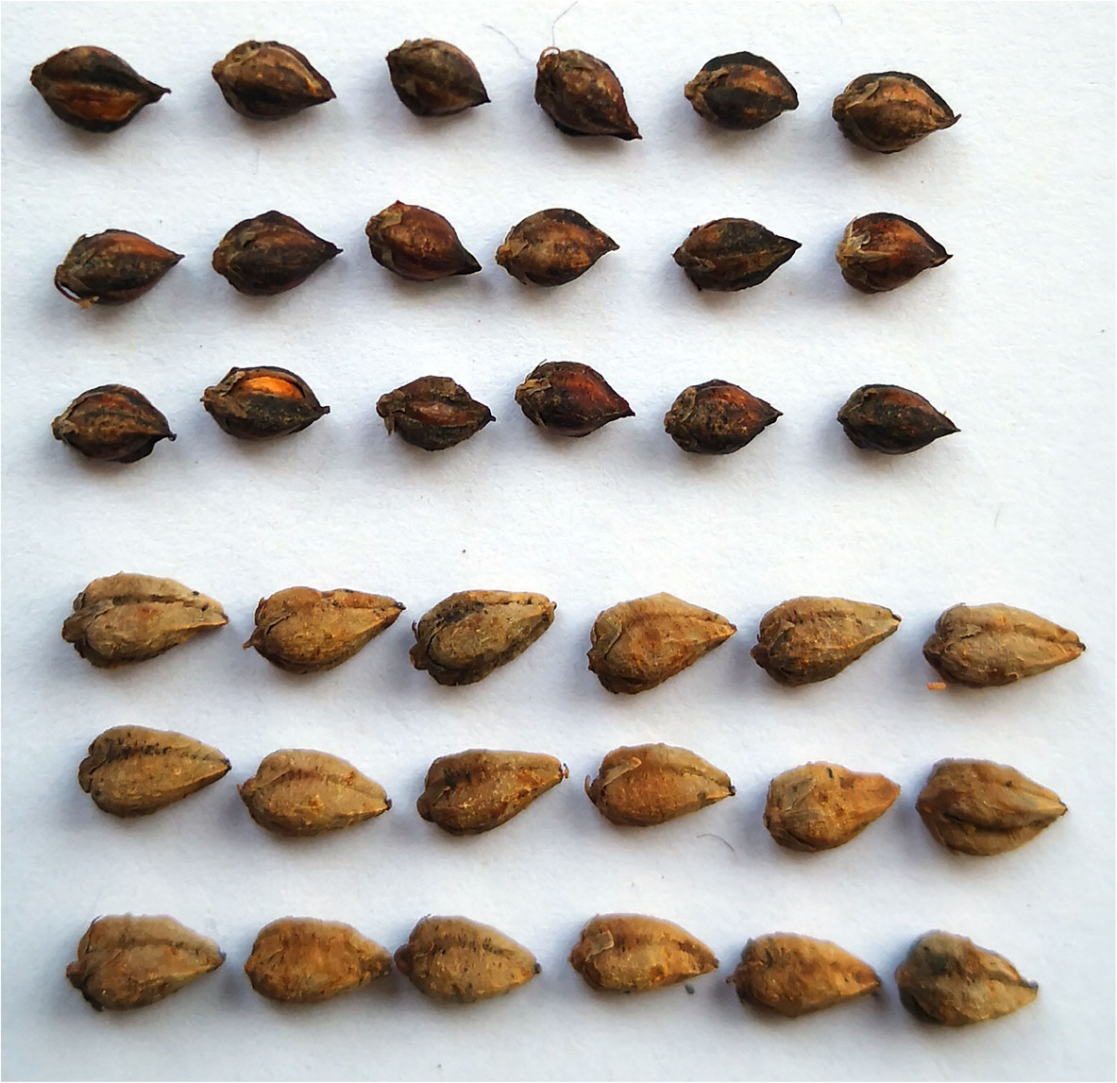
Grain samples of female (Xiaomiqiao) and male (Jinqiaomai 2)

The two parents differed significantly in hull type and grain size (Fig. 3). The female parent Xiaomiqiao is a Rice-Tartary type with a thin and loose hull and has splits on the sides of the grains. The male parent Jinqiaomai 2 is a common Tartary buckwheat type with a thick and tough hull. Individual lines of the F_8_ XJ-RILs population were classified as either the Rice-Tartary type or Tartary buckwheat type based on the hull phenotype. Among the 221 F_8_ lines, 79 lines were of the Rice-Tartary type and 142 lines were of the Tartary buckwheat type. Of the 142 lines belong to Tartary buckwheat type, 3 lines (R51, R88 and R92) exhibited the segregation of hull type in F_9_ under two environments.

**Fig. 4:**
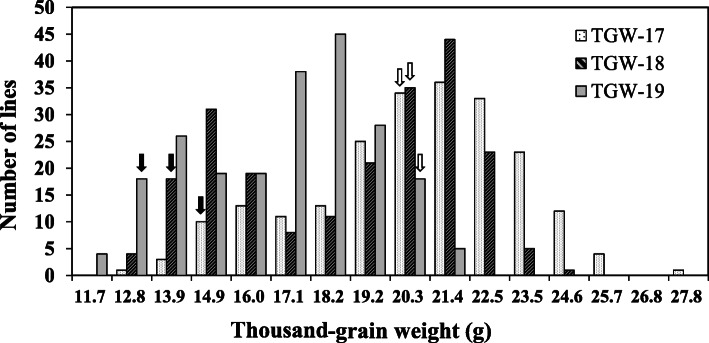
Frequency distribution of TGW in the XJ-RILs population derived from the cross of ‘Xiaomiqiao × Jinqiaomai 2’ under three field trials. The black and white arrows the values for the parents Xiaomiqiao and Jinqiaomai 2, respectively

TGW of Jinqiaomai 2 was greater than that of Xiaomiqiao in all three field trials (Table 3). The XJ-RILs population showed transgressive segregation and wide variation with the individual coefficients from 13.97 to 16.78% in the three field trials (Table 3). A bimodal distribution of thousand-grain weight was observed and similar distribution existed in all three field trails, indicating involvement of major genes (Fig. 4).

**Table 3:** Mean values and ranges of the TGW (g) in the parents and the XJ-RILs population

Environment	Parents		XJ-RILs population				
	Jinqiaomai 2	Xiaomiqiao	Mean	Range	CV%	Skewness	Kurtosis
2017	20.16 ± 0.57a	14.79 ± 0.87b	19.95	12.50–27.30	13.97	−0.46	− 0.25
2018	20.49 ± 1.14a	14.26 ± 2.69b	18.18	12.01–23.69	16.78	−0.32	−1.27
2019	20.40 ± 0.44a	12.34 ± 0.26b	16.28	11.17–21.12	14.76	−0.25	− 0.92

### QTLs detection for TGW

**Fig. 5:**
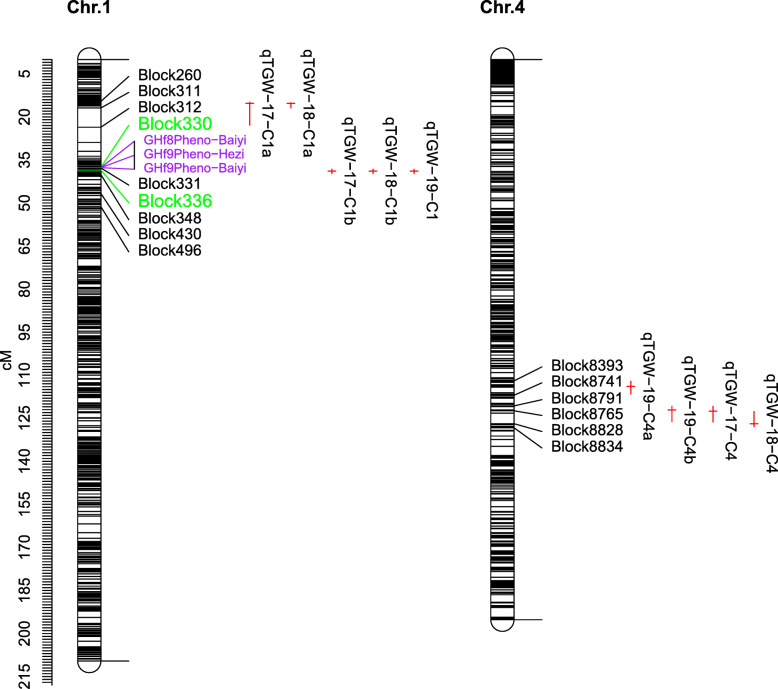
Locations of genes controlling hull type and identified QTLs for TGW in the XJ-RILs population derived from the cross of ‘Xiaomiqiao × Jinqiaomai 2’ under multiple environments. The markers marked in purple were phenotype markers of hull type from F_8_ and F_9_-RILs in two environments. TGW, 1000-grain weight. The red horizon lines indicated the peak position, and the red vertical lines indicated the confidence interval of QTLs

We used the high-density SNP linkage map to identify QTLs for TGW. In total, 9 QTLs affecting TGW were identified from all the three trials. These QTLs distributed on four loci on Chr.1 and Chr.4 (Table 4 and Fig. 5). One major locus detected in all three trials was mapped in the 38.2–39.8 cM region on Chr.1, with an LOD score of 18.1–37.0, and explained for 23.6–47.5% of the phenotypic variation. Two minor loci were repeatly detected in two or three trials. One was located in the 14.9–22.9 cM region on Chr.1 detected in both 2017 and 2018, accounting for 3.4 and 5.0% of the phenotypic variation, respectively. Another was mapped in 122.6–128.0 cM region on Chr. 4 detected in all three trials, explaining 3.1–10.9% of the phenotypic variation (Table 4 and Fig. 5).

**Table 4:** QTLs for TGW identified in XJ-RILs population of Tartary buckwheat in three field trials

QTL	Chr	Position (cM)	LOD	R^2^%	Additive effect	Confidence interval (cM)	Marker Interval
*q*TGW-17-C1a	1	15.31	4.4	5.0	− 0.64	14.9–22.9	Block260-Block312
*q*TGW-18-C1a	1	15.31	3.9	3.4	−0.58	15.1–16.9	Block260-Block311
*q*TGW-17-C1b	1	38.91	18.1	23.6	1.37	38.2–39.8	Block332-Block348
*q*TGW-18-C1b	1	38.91	37.0	47.5	2.15	38.2–39.8	Block332-Block348
*q*TGW-19-C1	1	38.91	18.2	24.4	1.21	38.2–39.8	Block332-Block348
*q*TGW-19-C4a	4	113.9	3.5	2.9	0.41	112.1–116.6	Block8393-Block8741
*q*TGW-19-C4b	4	122.1	8.3	6.6	0.63	120.8–126.2	Block8791-Block8828
qTGW-17-C4	4	122.5	13.3	10.9	0.93	120.8–126.3	Block8791-Block8828
*q*TGW-18-C4	4	126.9	5.3	3.1	0.54	122.6–128.0	Block8765-Block8834

### Mapping and identification of candidate gene controlling hull type

The phenotype markers for hull type in multiple environments were mapped to Chr.1 between marker Block330 and Block331 (Fig. 5). To identify candidate genes controlling hull type, the interval between marker Block330 and Block336 was mapped to a 40.9 kb region based on the Pinku1 Tartary buckwheat reference genome [24]. Seven genes were located in this region, six of which were annotated with the GO, COG, KEGG, KOG, Swiss-Prot and Nr databases (Table 5 and Additional file 6: Table S5).

**Table 5:** Annotation of the candidate genes controlling hull type

GeneID	Location	Direction	Annotation
*FtPinG0001417500.01*	6,437,204–6,438,135	+	Putative cell wall protein
*FtPinG0001417800.01*	6,438,913–6,441,059	–	Mpv17/PMP22 family
*FtPinG0001417900.01*	6,445,426–6,446,351	+	–
*FtPinG0001418200.01*	6,446,910–6,451,395	–	ABC-2 family transporter protein
*FtPinG0001418300.01*	6,455,603–6,462,172	+	Pentatricopeptide repeat-containing protein
*FtPinG0001418500.01*	6,466,630–6,467,517	+	Hypothetical protein
*FtPinG0001419000.01*	6,476,714–6,479,601	–	RING-variant domain

**Fig. 6:**
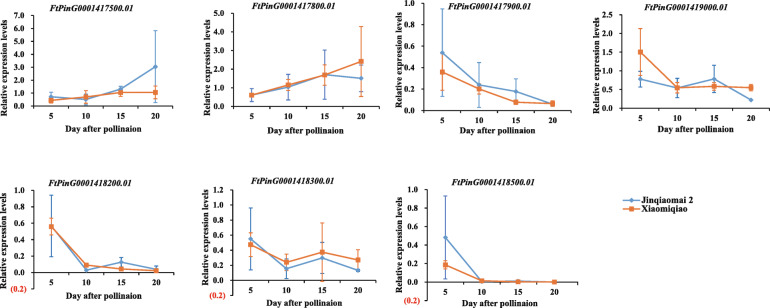
Expression patterns of candidate genes controlling hull type in Xiaomiqiao and Jinqiaomai 2

We analysed the expression patterns of the seven candidate genes in the parental plants Xiaomiqiao and Jinqiaomai 2. The expression levels of the seven candidate genes were low during seed development, and there were no significant differences in the expression levels of all the candidate genes between the two parents (Fig. 6). We then compared the sequences of the candidate genes between the two parents using the re-sequenced genome. A non-synonymous SNP was identified in *FtPinG0001417500.01*, two non-synonymous SNPs in *FtPinG0001418200.01*, two non-synonymous SNPs in *FtPinG0001418300.01* and a non-synonymous SNP *FtPinG0001418500.01* (Table 6).

**Table 6:** Annotation of SNPs located in the candidate genes controlling hull type

Gene ID	Transcript ID	Exon#	Mutation/ Nucleotide	Mutation/Protein
*FtPinG0001417500.01*	*FtPinG0001417500.01.T01*	exon1	c.A223T	p.T75S,Threonine; Serine
*FtPinG0001418200.01*	*FtPinG0001418200.01.T01*	exon8	c.A1560G	p.I520M,Isoleucine; Methionine
*FtPinG0001418200.01*	*FtPinG0001418200.01.T01*	exon6	c.C1108T	p.R370W,Arginine; Threonine
*FtPinG0001418300.01*	*FtPinG0001418300.01.T01*	exon9	c.G1787C	p.R596T, Arginine; Threonine
*FtPinG0001418500.01*	*FtPinG0001418500.01.T01*	exon3	c.G169A	p.D57N, Aspartic acid; Asparagine
*FtPinG0001418500.01*	*FtPinG0001418500.01.T01*	exon3	c.T183G	p.S61R, Threonine; Arginine

## Discussion

Genetic linkage maps are an important basis for genomic research, QTLs and qualitative traits loci mapping, marker-assisted breeding and map-based gene cloning of important genes. However, the construction of genetic linkage maps and QTLs mapping in Tartary buckwheat have remained limited, due mainly to the difficulty of hybridization in Tartary buckwheat to develop a mapping population and to the lack of genomic and genetic resources to identify enough markers for genotyping and QTLs analysis. Several genetic linkage maps from different populations have been constructed based on relatively few SSR markers in Tartary buckwheat [25, 26]. However, the marker density of these reported genetic maps was not enough to map QTLs for important agronomic characteristics.

In recent years, the rapid development of next-generation sequencing (NGS) technologies has greatly enriched genomic resources [27–30] and promoted the large-scale identification of molecular markers [31] and the construction of genetic maps and QTLs mapping of important traits in buckwheat. Yabe et al. [32] constructed a high-density genetic linkage map of common buckwheat using the DNA microarray method. The map consisted of 756 bin markers and contained 8884 SNPs distributed over 8 linkage groups with an average spacing of 2.13 cM between adjacent markers, and four QTLs for main stem length were mapped. Yasui et al. [33] published a common buckwheat reference genome and mined new candidate genes controlling the heteromorphic self-incompatibility of common buckwheat. The reference genome sequence of Tartary buckwheat was released recently [24], which promoted the development of large-scale molecular markers and the construction of a high-density genetic map. In this study, the XJ-RILs population consisting of 221 F_7_ lines developed from a cross between the Rice-Tartary cultivar Xiaomiqiao and Tartary buckwheat cultivar Jinqiaomai 2 was employed to construct the first high-density SNP genetic map of Tartary buckwheat based on RAD re-sequencing. The linkage map consisted of 4151 bin markers comprising 122,185 SNPs distributed on 8 linkage groups, covering 1444.15 cM with an average distance of 0.35 cM between adjacent markers. To our knowledge, this is the highest density genetic map of Tartary buckwheat. This high-density linkage map is expected to be a valuable resource for genomic analysis and fine-scale QTL mapping in Tartary buckwheat.

The XJ-RILs mapping population constructed in the study is a stable genetic population and can be planted in multiple environments to repeatedly test and identify the steady QTLs of target traits. In this study, QTLs for TGW were detected using the high-density SNP linkage map in three environments. Nine QTLs for TGW were detected and distributed on four loci on Chr.1 and Chr.4. A major and reliable locus was mapped in 38.2–39.8 cM region on Chr.1, which was detected in all three trials with an LOD score of 18.1–37.0, and explained for 23.6–47.5% of the phenotypic variation. Two minor and reliable loci were repeatly detected in two or three environments located in the 14.9–22.9 cM region on Chr. 1 and 122.6–128.0cM region on Chr. 4, respectively. These results were consistent with the bimodal distribution of TGW in the XJ-RILs population, indicating involvement of major genes. The identified locus will be valuable for gene cloning and for revealing the mechanism underlying grains development.

Rice-Tartary cultivars have increasingly received attention from researchers in recent years for their easy dehulling. It has been confirmed that the Rice-Tartary type is recessive to Tartary buckwheat type, and a single gene controls this character [13, 16–18]. However, the gene underlying easy dehulling had not been identified until now. In the present study, individual lines of the XJ-RILs population were investigated and classified as the Rice-Tartary type or Tartary buckwheat type based on the hull phenotype in multiple environments. The phenotype markers for hull type were used as molecular markers for genotyping and linkage grouping. Genes controlling hull type were mapped to Chr.1 between marker Block330 and Block331, which was closely followed by the major locus underlying TGW, indicating that the locus has pleiotropism or physiological association with TGW. To identify the candidate genes controlling hull type, the region between Block330 and Block336 was mapped to the Tartary buckwheat (Pinku1) reference genome, ranging from 6,428,375 to 6,469,300 bp and spanning 40,925 bp. Liu et al. [21] found that *FtpinG0009028000.01* gene has the potential effect on the cracking of Tartary buckwheat fruit, which was located on chromosome 7. Fukuie et al. [22] found that easy dehulling in Rice-Tartary cultivars was associated with a G → A substitution in *FtAG*, which was located in chromosome 1, ranging from 6,814,952 to 6,819,417 bp, and was approximately 350 kb downstream of the locus controlling hull type mapped in the present study. Zhang et al. [23] identified a genetic region underlying easy dehulling by combining BSA and high-throughput sequencing based on the reference genome of Tartary buckwheat, ranging from 5,999,388 to 6,856,630 bp and spanning 857,243 bp, and this region contained 45 high-impact SNPs/indels and 36 genes. The region included the locus controlling hull type mapped in the present study and *FtAG* identified by Fukuie et al. [22].

Seven candidate genes were located in the confidence interval of 6,814,952 to 6,819,417 bp. The expression levels of the seven candidate genes were low during grain development, and no significant difference was observed between the parental lines. We speculate that there may be three reasons for this result. First, the target gene was mapped in the interval, but the phenotypic differences in hull type may be mainly due to the variation in coding sequences of the target gene between the two parents. There are four candidate genes (*FtPinG0001417500.01*, *FtPinG0001418200.01*, *FtPinG0001418300.01* and *FtPinG0001418500.01*) with non-synonymous SNPs between the two parents, which may be associated with easy dehulling. Some of these non-synonymous SNPs may result in changes in the structure and function of the encoded protein, leading to differences in the target traits between the two parents. Second, the target gene is deleted from the region of the reference genome*.* The karyotype of Rice-Tartary has been reported to be distinct from that of Tartary buckwheat in both chromosome length and the number of submetacentric chromosomes [14]. It is reasonable to hypothesize that the gene for easy dehulling is present in the genome of Rice-Tartary buckwheat but is absent in the genome of Tartary buckwheat, as observed in Pingku 1 and Jinqiaomai 2, which resulted in the absence of the target gene in the relevant region of the reference genome. Third, the locus slightly deviates from the position of the target gene because of the error in genotyping, linkage construction and genes mapping, which may require an expansion of 100 kb, 200 kb or 500 kb from both flanks of the confidence interval to verify the candidate gene. Since there is no direct evidence regarding the candidate genes controlling the hull type, fine mapping of locus controlling hull type should be performed, the genomes of Xiaomiqiao and Jinqiaomai 2 need be de novo assembled for comparative genomic sequence analysis of the mapped region, and further experiments are required for functional validation of the differentially expressed genes between Rice-Tartary and Tartary buckwheat lines.

## Conclusion

In the present study, a high-density SNP map for Tartary buckwheat was constructed using a RILs population (Xiaomiqiao × Jinqiaomai 2) based on RAD sequencing. The high-density map consisted of 4151 bin markers comprising 122,185 SNPs, with an average distance of 0.35 cM between adjacent bin markers. To our knowledge, this is the highest density genetic map of Tartary buckwheat, which will be valuable for QTL mapping, gene identification, map-based gene cloning and comparative mapping in Tartary buckwheat. Furthermore, one major and reliable locus for TGW was identified using this map. Genes controlling hull type were mapped to Chr.1 between marker Block330 and Block331. In addition, four of the seven candidate genes located in the region were identified as having non-synonymous SNPs between the two parents that may be associated with easy dehulling. The present study provides important information for map-based cloning of genes underlying easy dehulling and grain development and the establishment of marker-assisted selection systems for breeding Tartary varieties with easy dehulling and large grains.

## Methods

### The mapping population

Xiaomiqiao is a local Rice-Tartary cultivar with thin and loose hull from Yunnan Province, while Jinqiaomai 2 is a Tartary buckwheat cultivar with thick and indehiscent hull bred by the Shanxi Academy of Agricultural Sciences Crops Institute. In our previous study, F_1_ hybrids from the cross of ‘Xiaomiqiao × Jinqiaomai 2’ showed thick and indehiscent hull, indicating the dominant inheritance. In F_2_ populations, the thick hull showed single dominant model, indicating the thin hull controlled by a recessive gene [17]. In the present study, an F_7_ recombinant inbred lines (XJ-RILs) population consisting of 221 lines was developed using a single seed descent method. The two parental lines and 221 F_7_ XJ-RILs were grown in fields at the Baiyi Experimental Station (N26°50′, E106°58′, 1146 m above sea level) of Guizhou Normal University (Guizhou Province, China) in August 2016. Individual lines were sown uniformly in a 2.0 m long row comprising 40 plants, with 0.05 m between plants and 0.33 m between rows. One plant was randomly selected from each RIL to extract DNA for RAD sequencing and test hull type of F_8_ after harvest. All RILs were harvested (F_7:8_ lines) separately in December 2016.

### DNA extraction and RAD sequencing

Genomic DNA was extracted from young leaves of each RIL and the two parents using the Tiangen Plant DNA Kit DP305 (Beijing, China) according to the manufacturer’s protocol. Genomic DNA quality determination, library construction and quality assessment were carried out by Biomarker Technology Co., Ltd. (Beijing, China) following the standard Illumina operating procedures as previously described [34]. The 221 RILs were sequenced by RAD, and the two parent lines were re-sequenced using the Illumina HiSeq2500 (Illumina, USA) platform.

### SNP genotyping and bin map construction

Raw reads obtained from the HiSeq2500 system were filtered to generate clean reads following the protocol described by Li et al. [34]. The clean reads from each sample were aligned onto the Pinku1 Tartary buckwheat reference genome [24] to estimate the distribution of insert size, calculate the sequencing depth and coverage ratio, and detect the genomic variation using BWA software [35]. Based on clean read alignment with the reference genome, duplicates were marked using the Picard software toolkit (http://sourceforge.net/projects/picard/), and local indel realignment and base recalibration were performed using the GATK software toolkit [36] to correct the base mass value. Then, SNPs were detected and filtered using GATK to obtain the final set of SNPs. The SNPs identified between the parents were considered polymorphic for subsequent bin calling. The 221 RILs were identified based on the parental SNP positions. SNPs were genotyped following the analytical approach described by Han et al. [37]. To guarantee the quality of the genetic map, bins less than 15 kb were initially eliminated, and bins with an extreme segregation distortion (*P* < 0.01) by the χ^2^ test were excluded.

The genetic map was constructed by high-quality bin markers using the HighMap program [38] following the protocol previously described by Hu et al. [39]. Spearman correlation coefficients were calculated using the genetic position and physical position of the bins directly to assess the collinearity between the genetic and physical maps.

### Measurement and QTLs mapping for TGW

Three replications for 221 F_7_:_8_ RILs and their parents were planted in a randomized plot design with each plot comprising three 2.0 m long rows, with 0.33 m between rows at the Hezi Experimental Station (N26°27′, E106°39′, 1066 m above sea level) of Guizhou Normal University in August 2017 and Baiyi Experimental Station in August 2018 and 2019, respectively. Each plot was harvested separately by hand at maturity stage to investigate TGW.

QTLs detection for TGW was performed using composite interval mapping (CIM) in WinQTL cartographer 2.5 software (http://statgen.ncsu.edu/qtlcar/ WQTLCart.htm). The QTL threshold (*P* < 0.05) was estimated from 1000 permutations. Each QTL was denominated as “*q*” (abbreviation of QTL) + trait name + environment + chromosome name + the serial letter. For example, *q*TGW-17-C1a and *q*TGW-17-C1b denote two QTLs for TGW detected on chromosome 1 in 2017.

### Hull type test, genes mapping and candidate genes prediction

Individual F_8_ XJ-RILs were classified as either the Rice-Tartary type or Tartary buckwheat type based on the hull phenotype, and the phenotype markers for hull type was denominated as ‘GHf8Pheno-Baiyi’. The F_8_ XJ-RILs and parents were planted to test the hull type in the same manner as F_7_ at the Hezi Experimental Station of Guizhou Normal University in August 2017 and Baiyi Experimental Station in August 2018. At the maturity stage, the hull type of all the representative individuals from each RIL were investigated, and the phenotype markers for hull type was denominated as ‘GHf9Pheno-Baiyi’ and ‘GHf9Pheno-Hezi’, respectively. These phenotype markers were used as molecular markers for genotyping and superimposed on the high-density SNP linkage map using JoinMap4.0 [40] according the method described by Li et al. [41]. The mapped region included phenotype markers was aligned onto the Pinku1 Tartary buckwheat reference genome. Candidate genes controlling hull type were analysed using annotations from the Pinku1 Tartary buckwheat reference genome [24].

### RNA isolation, cDNA preparation, and qRT-PCR analysis of candidate genes

Grains from Xiaomiqiao and Jinqiaomai 2 after pollination (5, 10, 15 and 20 days) were sampled and immediately frozen in liquid nitrogen and stored at − 80 °C. Samples from each stage consisted of three biological replicates. Total RNA was isolated from grains using the Tiangen Plant RNA Purification Kit (Beijing, China) according to the manufacturer’s instructions. RNA samples with an A260/A230 ratio ≥ 2.0 and A260/A280 ratio ≥ 1.8 were used to synthesize cDNA using the TAKARA PrimeScript™ RT reagent Kit with gDNA Eraser (Perfect Real Time) (Dalian, China).

Quantitative RT-PCR was performed using the CFX96TM Real-Time PCR Detection System (Bio-Rad, USA). The PCRs (20 μL) were conducted according to the instructions for the TB Green® *Premix Ex Taq™* II Kit (Tli RNaseH Plus) (RR820b, TAKARA, Dalian, China). All PCRs were performed in two biological replicates. The quantitative PCR conditions were as follows: 95 °C for 30 s, followed by 40 cycles of 95 °C for 5 s, 60 °C for 34 s, and 72 °C for 20 s. Data were analysed by the 2^−(ΔΔCt)^ method to obtain relative mRNA expression data. Primers used for qRT-PCR amplification of the candidate genes for hull type and of *actin* in Tartary buckwheat were designed using Primer Premier 6.0 and are listed in Additional file 7: Table S6.

## Supplementary Information

**Additional file 1: Table S1.** The summaries of high-throughput sequencing data.

**Additional file 2 Table S2.** Details regarding the SNPs in the parents and 221 RILs.

**Additional file 3: Figure S1.** Graphic representation of the genotypes of 221 RILs that were identified using a sliding window approach along each chromosome.

**Additional file 4: Table S3.** The genotype of all the bin markers mapped on the genetic map.

**Additional file 5: Table S4.** The blocks and their physical distance in the genetic map.

**Additional file 6: Table S5.** Annotation of the candidate genes controlling hull type with the GO, COG, KEGG, KOG, Swiss-Prot and Nr databases.

**Additional file 7: Table S6.** The primers sequence of candidate genes controlling hull type and *actine* gene used in this study.

## Data Availability

The data supporting the results presented in this article are included as additional files. The Pinku1 Tartary buckwheat reference genome sequence and the gene annotation version 2 used in the study can be retrieved from http://www.mbkbase.org/Pinku1/. The raw sequencing data of the two parents and the 221 RILs was deposited in the National Genomics Data Center Genome Sequence Archive (GSA) database (https://bigd.big.ac.cn/bioproject/), Beijing Institute of Genomics (BIG), Chinese Academy of Sciences, and are publicly available under the BioProject accession number PRJCA003285 in https://bigd.big.ac.cn/bioproject/

## Abbreviations

RAD: Restriction site-associated DNA
*F.tataricum*: *Fagopyrum tataricum*
*F. esculentum*: *Fagopyrum esculentum*
QTLs: Quantitative trait loci
SNP: Single-nucleotide polymorphism
RIL: Recombinant inbred line
SSR: Simple sequence repeat
NGS: Next-generation sequencing
TGW: 1000-grain weight
