# Supplementary figures and images for: Mapping QTLs for 1000-grain weight and genes controlling hull type using SNP marker in Tartary buckwheat (Fagopyrum tataricum)
Source: BMC Genomics. 2021 Feb 27;22:142. doi: 10.1186/s12864-021-07449-w (PMC7913328; doi:10.1186/s12864-021-07449-w)

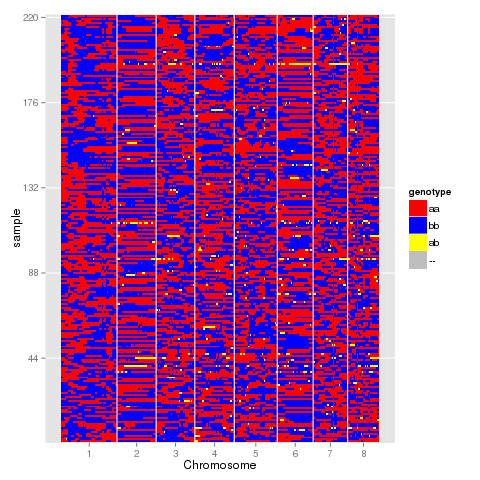

Supplement: Supplementary file 3 — Additional file 3: Figure S1. Graphic representation of the genotypes of 221 RILs that were identified using a sliding window approach along each chromosome. [file 12864_2021_7449_MOESM3_ESM.png]
