# Supplementary material for: Mapping QTLs for 1000-grain weight and genes controlling hull type using SNP marker in Tartary buckwheat (Fagopyrum tataricum)
Source: BMC Genomics. 2021 Feb 27;22:142. doi: 10.1186/s12864-021-07449-w (PMC7913328; doi:10.1186/s12864-021-07449-w)
Supplement: Supplementary file 7 — Additional file 7: Table S6. The primers sequence of candidate genes controlling hull type and actine gene used in this study. [file 12864_2021_7449_MOESM7_ESM.docx]

Table S6 The primers sequence of candidate genes controlling hull type and *actine* gene used in this study

| Gene | Forward primer 5'-3' | Reverse primer 5'-3' |
| --- | --- | --- |
| *FtPinG0001417500.01* | CGGTTCGTCTGTTCCTACTT | TTATGGGTTGGAAGGGATGG |
| *FtPinG0001417800.01* | GAGAATCACCAGCCAACATC | GTTACCCAGATGAGTTCCAC |
| *FtPinG0001417900.01* | GGTGATGATTCTCCTTCTCC | ACAACCACCGCAATAACACC |
| *FtPinG0001418200.01* | ACTGGCTCTCTCTGCATTTC | TGGAAAGAGGTGGGATTAGG |
| *FtPinG0001418300.01* | GGTAGAGTTGAAGATGGCAG | TTCCCTTTTATGTGGCCTCG |
| *FtPinG0001418500.01* | ACACAGATTCCCCAAGATCG | CTTCCCTCACATTCTCACTG |
| *FtPinG0001419000.01* | GTTCGCACTGTTGGTTCTCT | CGTCTCCTTCTCCATCTAAG |
| *FtActin* | ATGTTCACTACCACCGCTGA | TGAACCTCTCAGCACCAATC |
